# A Rare Case of Mucinous Adenocarcinoma of the Colon Presenting as Ileoileal Intussusception in an Adult

**DOI:** 10.1155/2012/340947

**Published:** 2012-02-12

**Authors:** Prakash Chand, Avani A. Patel, Kelly L. Cervellione, Muhammed Sulh

**Affiliations:** ^1^Department of Medicine, Jamaica Hospital Medical Center, 8900 Van Wyck Expressway, Jamaica, NY 11418, USA; ^2^Department of Clinical Research, Jamaica Hospital Medical Center, 8900 Van Wyck Expressway, Jamaica, NY 11418, USA; ^3^Department of Pathology, Jamaica Hospital Medical Center, 8900 Van Wyck Expressway, Jamaica, NY 11418, USA

## Abstract

Intussusception is the cause of around 1% of all bowel obstructions in adults. Unlike in children, where intussusception is most often idiopathic in nature, cases in adults usually have an identifiable etiology, most commonly malignancy. Symptoms are usually non-specific, but timely identification and management is crucial due to high rates of carcinoma as the lead point of intussusception. Here we present a rare case of mucinous adenocarcinoma of the colon that presented as ileoileal intussusception. Diagnostic and treatment issues are also discussed.

## 1. Introduction

Intussusception occurs when proximal bowel segment (intussusceptum) invaginates into distal bowel segment (intussuscipiens). This may cause a loss of blood supply to the area and intestinal obstruction. More rarely, the intussusceptum may become strangulated, necrotic, and gangrenous and lead to sepsis or death. Intussusception is a rare occurrence in adults, representing less than 5% of all cases of intussusception [1]. Intussusception is the cause of around 1% of all cases of bowel obstructions in adults [2]. Whereas intussusception in children is often idiopathic in nature, in adults there is more often a lead point and is caused by an underlying condition [3]. Reports have identified malignant tumors as the cause of intussusceptions in 65% to 87% of all adult cases [3–5]. Clinically, intussusception in adults often presents with nonspecific symptoms such as abdominal pain, nausea, diarrhea, and rectal bleeding. The classical triad of symptoms seen in children of sausage-shaped palpable mass, red currant jelly stools, and acute abdominal pain is less often seen in adults [2, 6]. Due to the high frequency of occurrence of intussusception secondary to malignancy in adults, surgical resection is usually indicated. Here we present a rare case of colon carcinoma in a young adult presenting as intestinal obstruction secondary to ileoileal intussusception.

## 2. Case Report

**Figure 1:**
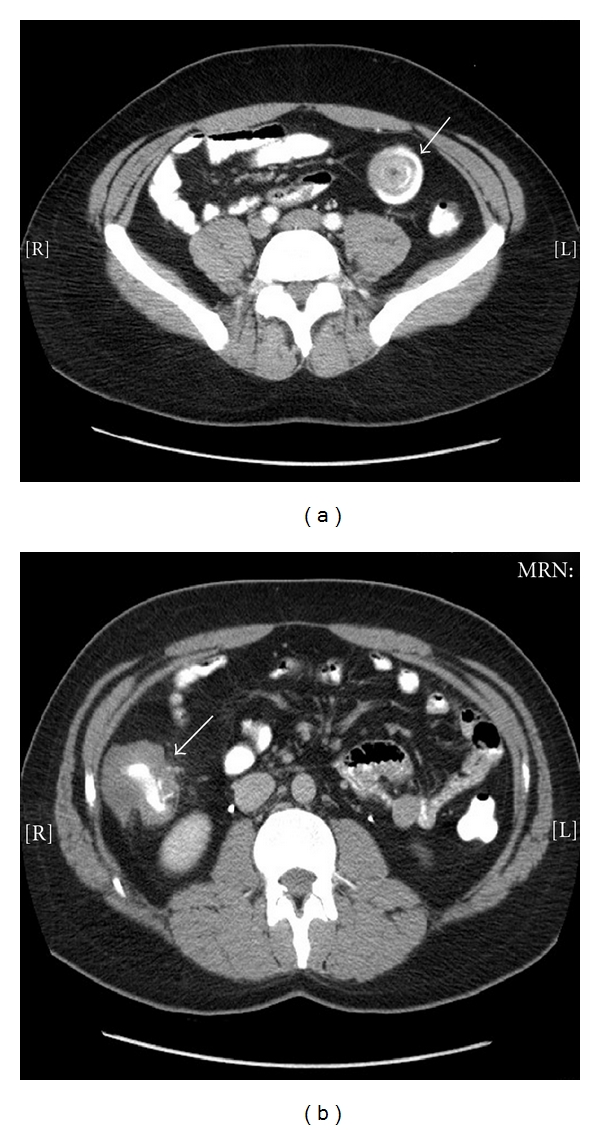
CT scan revealed intussusception of the small bowel (a) and thickening of the right colon (b).

A 29-year-old Hispanic male presented to the medicine clinic with episodes of pain in the abdomen for 10 months. Initially the pain was in the upper abdomen and infrequent, but later progressed to the lower abdomen and became more severe, intolerable, frequent, and prolonged, lasting about 15–20 minutes per occurrence. Associated symptoms were alteration in bowel movements, loss of appetite, and unintentional weight loss of about 5 pounds in 5 months. He reported 2 episodes of rectal bleeding before coming to the clinic. He denied any vomiting or fever. Physical examination revealed deep epigastric and right upper quadrant tenderness with no rebound tenderness, guarding, or abdominal distension. There was no palpable mass. On rectal exam, vault was empty and guaiac test was negative. CT scan of abdomen showed intussusception of small bowel and thickening of right colon (Figure 1). A few lymph nodes were seen in fat adjacent to the right colon and mesentery. He was sent to the emergency department and was admitted to surgery for further evaluation and management.

**Figure 2:**
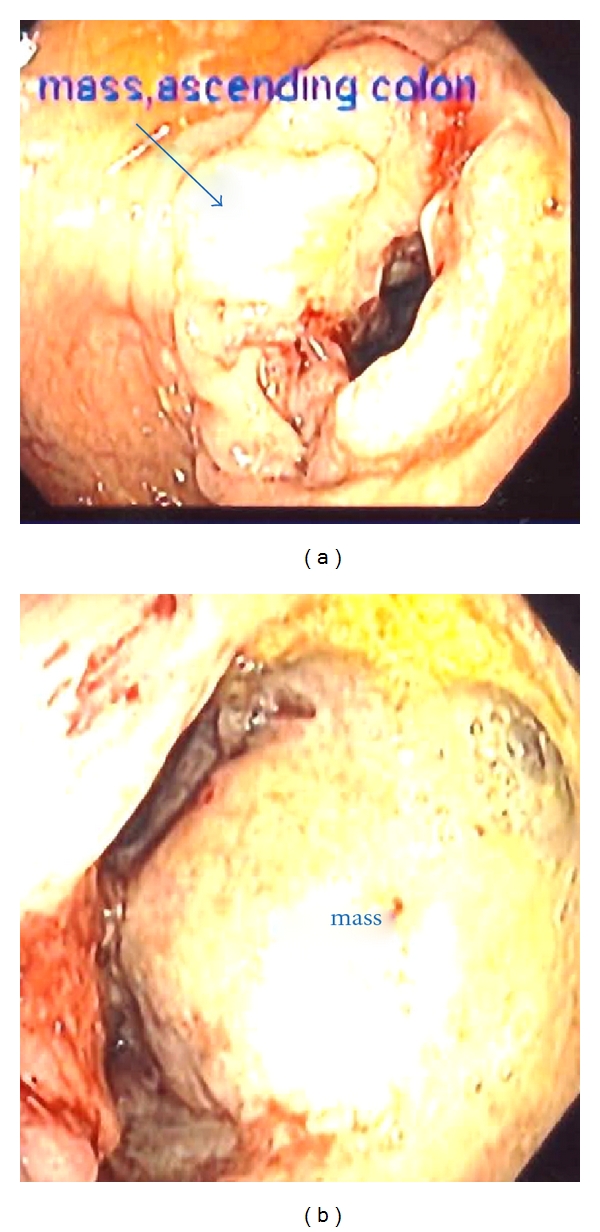
Colonoscopy revealed a circumferential fungating mass in the ascending colon measuring 4 cm in length.

**Figure 3:**
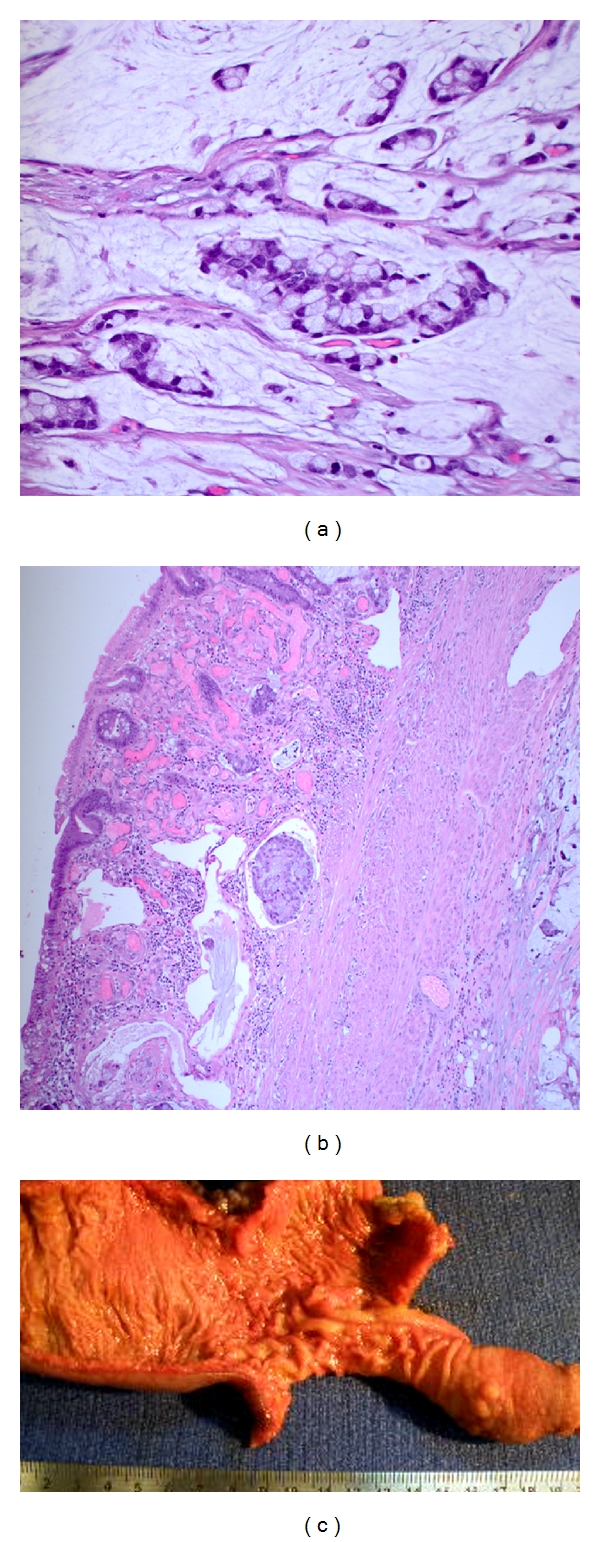
Histopathology of colon biopsy at high power field reveals malignant cells (a). Histopathology of ileum biopsy reveals metastatic malignancy (b). Gross histology revealed a metastatic ileal deposit as a lead point of intussusception (c).

Subsequently, colonoscopy revealed a circumferential fungating mass in the ascending colon measuring 4 cm in length (see Figure 2). Biopsies of the specimen showed moderate to poorly differentiated mucinous adenocarcinoma. Patient underwent exploratory laparotomy. Intraoperative findings were diffuse peritoneal and mesenteric metastatic studding, large extraluminal right colon cancer, and ileoileal intussusception in midileum with lead point metastatic deposit (see Figure 3). Thirty centimeters of ileum was resected with ileoileal side-to-side anastomosis, and right hemicolectomy with side-to-side ileocolic anastomosis was performed. The patient was discharged after 6 days. The surgical pathology was reported as moderate to poorly differentiated signet ring cell mucinous adenocarcinoma in proximal ascending colon with serosal and lymphovascular invasion (see Figure 3). There was also metastatic mucinous adenocarcinoma as a lead point of ileoileal intussusception and omental metastasis. Stage IV A (pT4b pN2 PM1) cancer was diagnosed based on all findings. The patient was followed in clinic after five weeks and was asymptomatic with good healing of wound.

## 3. Discussion

 Adult intussusception is a rare condition representing the cause of approximately 0.003% (3 of every 100,000) of hospital admissions in this population [7]. The diagnosis of adult intussusception is difficult due to the varied, nonspecific symptoms upon presentation [2, 6]. Whereas acute abdominal pain is common in children, adults with intussusception usually report a history of intermittent abdominal pain over a long period of time. Vomiting, nausea, and hematochezia are also common symptoms in adults [4, 8]. Bleeding is more frequent in colonic than in ileal intussusceptions. Unlike in pediatric populations, where a palpable, sausage-shaped mass can often be felt on physical examination, abdominal masses are palpable in as few as 10% of cases in adults [2, 6]. As in our patient, enteric intussusception often presents as abdominal pain, nausea, and vomiting. Colonic intussusception may present with constipation, lower GI bleed, and loss of weight.

Whereas intussusception in pediatric patients is usually idiopathic in nature, fewer than 10% of cases in adults are due to an unknown cause [9]. In adults, studies have shown that intussusception is caused by malignant tumors in up to 87% of cases, depending on the site of the lead point, with most others being caused by benign tumors [3–5]. The highest rates of malignancy are in the colon, whereas malignancy in enteric intussusception is less frequent [10].

Correct preoperative diagnosis is made in less than 50% of cases of adult intussusception [2]. Likelihood of correct diagnosis increases with the use of radiological tests, especially CT scans [2, 4, 11]. The classical findings on abdominal CT scans are target signs or doughnut signs on cross-section and pseudokidney signs on coronal sections [12]. In our case, like previous reports [11], we were able to diagnose ileoileal intussusception preoperatively using CT. Barium studies and ultrasonography may also aid in diagnosis. Colonoscopy is useful when presenting symptoms indicate colonic obstruction or CT scan points to colonic pathology [13].

There is some controversy surrounding the most appropriate methods for management of adult intussusception. Depending on the location, etiology, suspicion of malignancy, and clinical condition of the patient, several methods could be utilized. However, in adults, surgical resection of the lesion is most often indicated, especially in colonic intussusception given the high likelihood of malignancy. In some cases, reduction of the lesion prior to resection may be performed. In enteric intussusception, operative reduction may be attempted if the bowel is not ischemic or friable and malignant lesion is not suspected [4, 9, 14]. In general, prognosis in adult patients with intussusception due to nonmalignant causes is good. Unfortunately, since a vast number of patients develop intussusceptions secondary to malignant lesions, the overall survival rate in this population is adversely affected. Mortality due to intussusception in adult cases where the lead point is a benign lesion is less than 10%, whereas mortality in cases where malignancy is the causative factor is over 50% [2]. In our patient, even after extensive surgical resection with right hemicolectomy and resection of ileal intussusception, prognosis is still poor because of advanced disease, poor differentiation, and signet ring cell histology.

In conclusion, adult intussusception is a rare and challenging entity for clinicians due to the nonspecific symptoms that accompany the diagnosis. CT scan of the abdomen is the most useful and less invasive tool for diagnosis. Operative resection without reduction is the preferred treatment in adults as there is high risk of underlying malignancy.
